# Supplementary figures and images for: Risk Allele Frequency Analysis and Risk Prediction of Single-Nucleotide Polymorphisms for Prostate Cancer
Source: Genes (Basel). 2022 Nov 5;13(11):2039. doi: 10.3390/genes13112039 (PMC9689911; doi:10.3390/genes13112039)

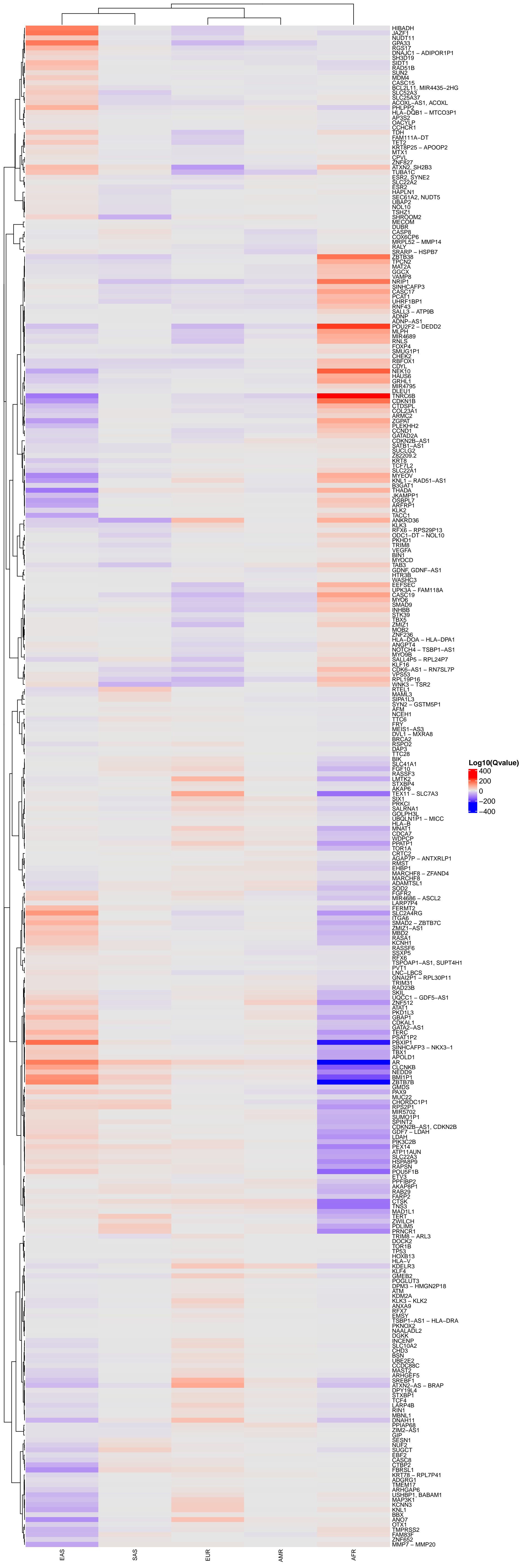

Supplement: Supplementary file 1 [file genes-13-02039-s001.zip › FigureS1.pdf]

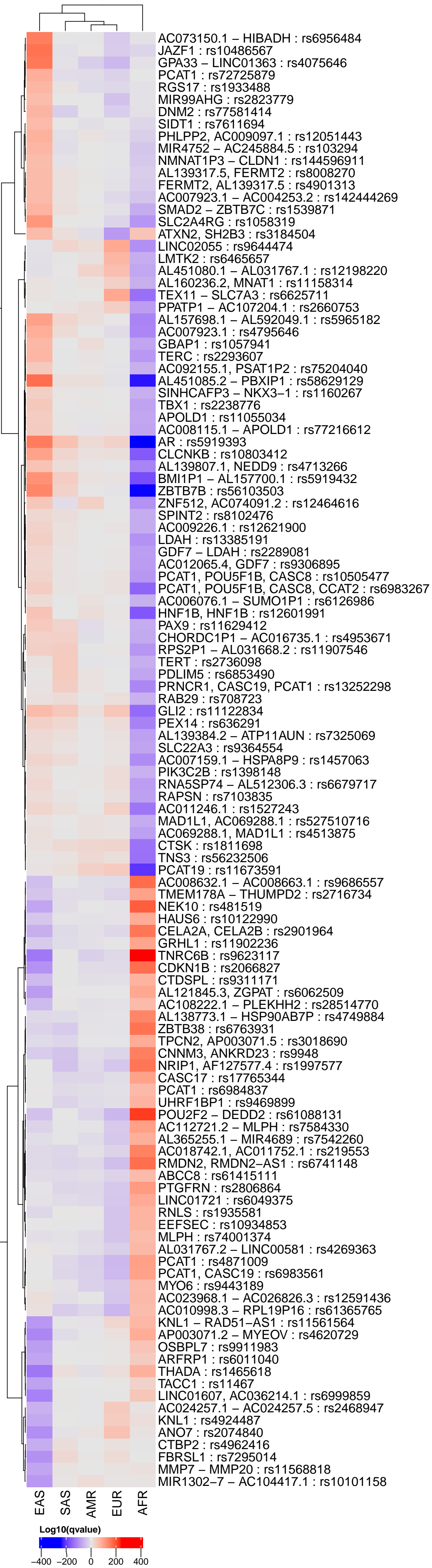

Supplement: Supplementary file 1 [file genes-13-02039-s001.zip › FigureS2.pdf]

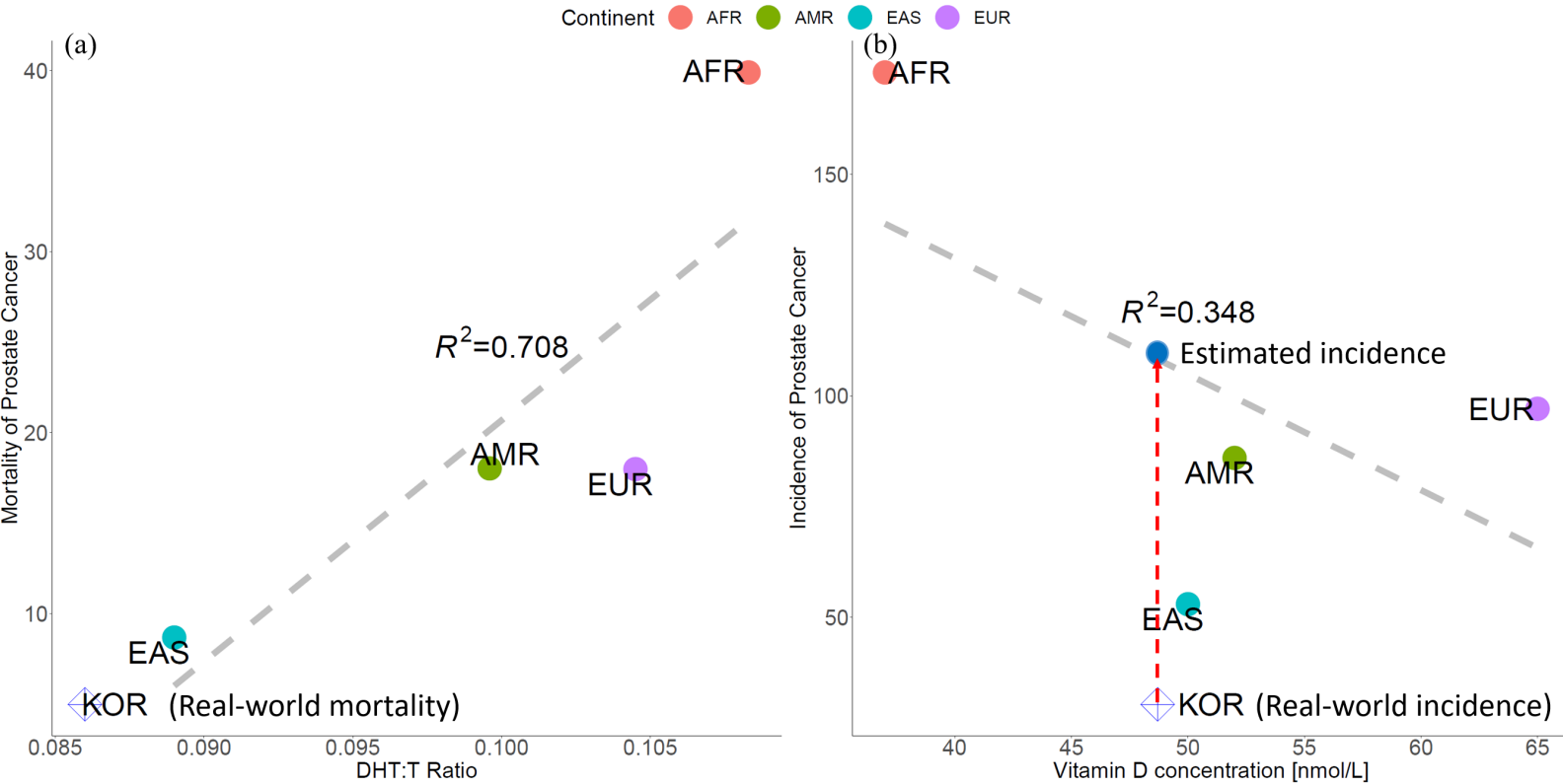

Supplement: Supplementary file 1 [file genes-13-02039-s001.zip › FigureS3.pdf]

# PROSTATE CANCER

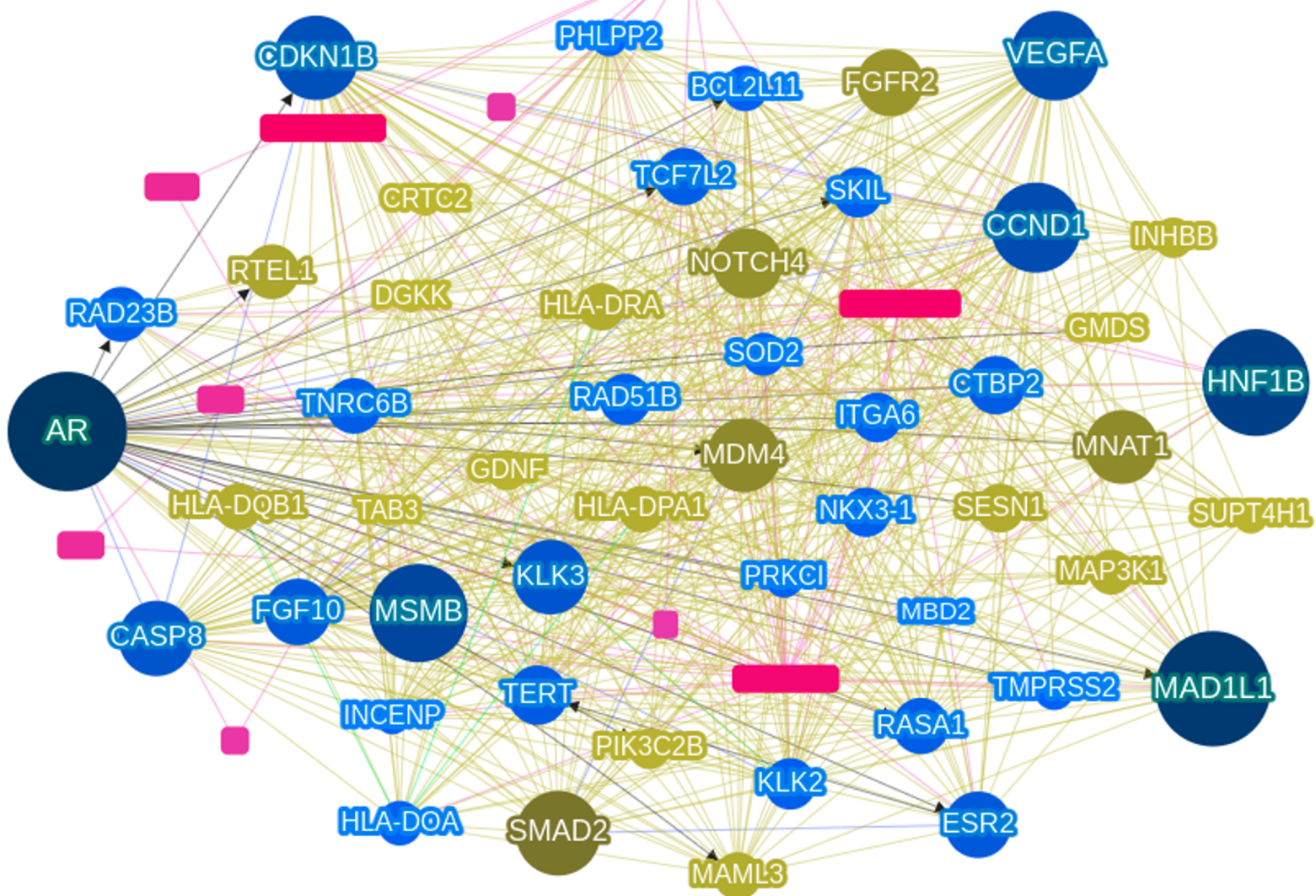

Supplement: Supplementary file 1 [file genes-13-02039-s001.zip › FigureS4.pdf]
